# Association between polymorphisms in DNA repair genes and survival of non-smoking female patients with lung adenocarcinoma

**DOI:** 10.1186/1471-2407-9-439

**Published:** 2009-12-15

**Authors:** Zhihua Yin, Baosen Zhou, Qincheng He, Mingchuan Li, Peng Guan, Xuelian Li, Zeshi Cui, Xiaoxia Xue, Meng Su, Rui Ma, Weijun Bai, Shuyue Xia, Yanduo Jiang, Shun Xu, Yi Lv, Xun Li

**Affiliations:** 1 Department of Epidemiology, School of Public Health, China Medical University, Shenyang 110001, PR China; 2 Key Laboratory of Cancer Etiology and Intervention, University of Liaoning Province, Shenyang 110001, PR China; 3 The Third Center for Laboratory Technology and Experimental Medicine, China Medical University, Shenyang 110001, PR China; 4 Department of Internal Medicine, Liaoning Cancer Hospital & Institute, Shenyang 110042, PR China; 5 Department of Respiratory Medicine, Fengtian Hospital Affiliated to Shenyang Medical College, Shenyang 110024, PR China; 6 Department of Pathology, 202 Hospital of Chinese PLA, Shenyang 110003, PR China; 7 Department of Thoracic Surgery, The First Affiliated Hospital of China Medical University, Shenyang 110001, PR China; 8 Shenyang Center for Disease Control and Prevention, Shenyang 110031, PR China

## Abstract

**Background:**

Excision repair cross-complementing group 1 (ERCC1) and group 2 (ERCC2), and X-ray repair cross-complementing group 1 (XRCC1) proteins play important roles in the repair of DNA damage and adducts. Single nucleotide polymorphisms (SNPs) of DNA repair genes are suspected to influence treatment effect and survival of cancer patients. This study aimed to investigate the relationship between polymorphisms in *ERCC2*, *ERCC1 *and *XRCC1 *genes and survival of non-smoking female patients with lung adenocarcinoma.

**Methods:**

We used polymerase chain reaction-restriction fragment length polymorphism (PCR-RFLP) method to evaluate SNPs in *ERCC2*, *ERCC1 *and *XRCC1 *genes among 257 patients.

**Results:**

The overall median survival time (MST) was 13.07 months. Increasing numbers of either *ERCC1 *118 or *XRCC1 *399 variant alleles were associated with shorter survival of non-smoking female lung adenocarcinoma patients (Log-rank P < 0.001). The adjusted hazard ratios (HRs) for individuals with CT or TT genotype at *ERCC1 *Asn118Asn were 1.48 and 2.67 compared with those with CC genotype. For polymorphism of *XRCC1 *399, the HRs were 1.28 and 2.68 for GA and AA genotype. When variant alleles across both polymorphisms were combined to analysis, the increasing number of variant alleles was associated with decreasing overall survival. Using the stepwise Cox regression analysis, we found that the polymorphisms in *ERCC1 *and *XRCC1*, tumor stage and chemotherapy or radiotherapy status independently predicted overall survival of non-smoking female patients with lung adenocarcinoma.

**Conclusions:**

Genetic polymorphisms in *ERCC1 *and *XRCC1 *genes might be prognostic factors in non-smoking female patients with lung adenocarcinoma.

## Background

Lung cancer is a major cause of cancer mortality worldwide, and more than a million people in the world die from the disease each year [1]. Adenocarcinoma accounts for about 25% to 40% of all lung cancers, and is now the most common form of lung cancer in women [2]. It is the most frequent subtype occurring in those who have never smoked. Five-year survival rate of lung cancer is at only 15% in the United States and even lower in China [3]. Genetic factors are considered to influence the treatment effectiveness of lung cancer [4], and thus affect the prognosis of patients. There are some molecular markers showing potential as therapeutic and prognostic indicators, but none could be used into clinical practice [5,6]. Of these factors, DNA repair capacity (DRC) is an important one. Several studies have shown associations between inefficient DNA repair and lung cancer risk [7,8]. It is also possible that individual DRC can affect the survival of lung cancer patients. It has been speculated that single nucleotide polymorphisms (SNPs) in DNA repair genes may change gene expression and activity, hence influence the effectiveness of cancer treatment and survival of patients [9]. To test above possibility, we assess the relationship between survival of lung adenocarcinoma patients and SNPs in three DNA repair genes, including excision repair cross-complementing group 1 (*ERCC1*) and group 2 (*ERCC2*), and X-ray repair cross-complementing group 1 (*XRCC1*). *ERCC2 *is located in chromosome 19q13.2-13.3 and codes for an evolutionarily conserved helicase, a subunit of TFIIH complex which is essential for transcription and nucleotide excision repair (NER). The common SNPs of *ERCC2 *gene is at codon 751 (A > C substitution at nucleotide position 35931, exon 23, Lys>Gln, rs13181) and codon 312 (G >A substitution at position 23951, exon 10, Asp>Asn, rs1799793). *ERCC1 *is also located in chromosome 19q13.2-13.3 and codes for a leading protein in NER, responsible for recognition of DNA damage and removal of the damaged nucleotides. The common SNP of *ERCC1 *gene is at codon 118 (C > T substitution at exon 4, without amino acid change--Asn/Asn, rs11615). The study showed that *ERCC1 *and *ERCC2 *mRNA levels were correlated with DRC [10]. XRCC1 protein plays a central role in base excision repair (BER) pathway by interacting with other DNA repair proteins. The most extensively studied SNP of *XRCC1 *gene is at codon 399 (G > A substitution at position 28152, exon 10, Arg>Gln, rs25487), which has been reported to be associated with an altered DNA repair activity [11,12]. SNP analyzing involves little more than a blood sample and relatively simple and precise polymerase chain reaction (PCR)-based techniques, making it more practical in the clinical testing than many other studied prognostic markers. Therefore, in this study we prospectively assess the relationship between the four SNPs in DNA repair genes and survival of non-smoking female patients with lung adenocarcinoma.

## Methods

### Patient recruitment and follow-up

All patients were from the ongoing study of lung cancer in non-smoking females, which started from July, 1999 in Shenyang city, China. The human investigations were approved by the Institutional Review Board of China Medical University, and informed consent was obtained from each participant or their representatives if direct consent could not be obtained. All patients were unrelated ethnic Han Chinese. Individual with a total of 100 cigarettes in his lifetime was defined as a smoker, otherwise he was considered as a non-smoker. Each participant donated 10 ml venous blood and was interviewed to collect demographic data and clinical information. For this study, we identified 285 patients who were diagnosed with histologically confirmed lung adenocarcinoma (stage I-IV) between the years 1999 and 2004. The year 2004 was chosen as the last year of eligibility in order that all participants have adequate follow-up. About the histological subtype of 285 patients, 228 were diagnosed using surgical specimen and 57 using exfoliated cells of lung adenocarcinoma patients whose tumor stages were determined by thoracic imageology.

Dates of death were obtained using at least one of the four following methods: inpatient and outpatient medical records, the registry of causes of death in Shenyang Center for Disease Control and Prevention (CDC), registration and presumption of death in Shenyang Public Security Bureau, and telephone follow-up. Twenty eight patients were lost to follow up. However, there was no significant difference in the characteristics between the patients with and without follow-up information. Finally, 257 patients were included in this analysis.

### DNA extraction and genotyping

Genomic DNA samples were extracted by guanidine hydrochloride (GuHCl) method. SNP was analyzed by polymerase chain reaction-restriction fragment length polymorphism (PCR-RFLP) method as described previously [13]. The PCR primers (Takara Biotechnology Dalian Co. Ltd., China) for amplifying DNA fragment containing the *ERCC2 *751, *ERCC2 *312, *ERCC1 *118 and *XRCC1 *399 sites were 751 F5'-GCC CGC TCT GGA TTA TAC G-3' and R5'-CTA TCA TCT CCT GGC CCC C-3', 312 F5'-CTG TTG GTG GGT GCC CGT ATC TGT TGG TCT-3' and R5'-TAA TAT CGG GGC TCA CCC TGC AGC ACT TCC T-3', 118 F5'-AGG ACC ACA GGA CAC GCA GA-3' and R5'-CAT AGA ACA GTC CAG AAC AC-3', 399 F5'-TTG TGC TTT CTC TGT GTC CA-3' and R5'-TCC TCC AGC CTT TTC TGA TA-3', respectively. The PCR products were digested with restriction enzyme (New England Biolabs, Beverly, MA) PstI (for *ERCC2 *751), StyI (for *ERCC2 *312), BsrdI (for *ERCC1 *118) and MspI (for *XRCC1 *399) to determine the genotypes. A 10% masked and random sample of patients was tested twice by different persons, and the results were found to be concordant for all of the masked duplicate sets.

### Statistical analysis

The associations between overall survival and demographic characteristics, clinical features, and genetic SNPs were estimated using the Kaplan-Meier method and Log-rank test. Survival time was calculated from the date of cancer diagnosis to the date of death or last follow-up. In some analyses, we combined the heterozygous genotype with the homozygous rare genotype as the particular group in order to increase sample size. Univariate and multivariable Cox proportional hazards regression models were performed to estimate crude hazard ratio (HR) or adjusted HR and their 95% confidence intervals (CIs). The stepwise Cox regression model was also used to determine factors predictive of cancer prognosis, with a significant level of P < 0.05 for entering and P > 0.10 for removal of the variables. All the statistical analyses were performed using Statistical Product and Service Solutions (SPSS) v13.0.

## Results

### Patient characteristics

The mean age of patients was 51.16 ± 9.48 years (range 18-75 years). The distribution of characteristics and clinical features of 257 lung adenocarcinoma patients were shown in Table 1. There were 206 deaths. The overall median survival time (MST) was 13.07 months. As Table 1 showed, patients with advanced cancer or without surgical operation had significantly shorter MSTs (Log-rank P < 0.05). Univariate Cox regression analysis suggested that the risks of death of lung adenocarcinoma were increased in patients with stage II, III and IV compared with those with stage I (HRs were 2.48, 3.46 and 4.66, respectively). The result also showed that the patients undergoing surgical operation had a decreased risk of death (HR = 0.61).

**Table 1:** Patient characteristics and clinical features

Variable	Patients (%)	MST(mon)	Log-rank P value	HR (95%CI)
Age (years)				
≤50	105(40.9)	14.93	0.493	1.00
>50	152(59.1)	12.47		1.10(0.84-1.45)
Tumor stage				
I	44(17.1)	45.80		1.00
II	34(13.2)	18.57	< 0.001	2.48(1.36-4.53)
III	149(58.0)	11.07		3.46(2.14-5.61)
IV	30(11.7)	9.07		4.66(2.59-8.37)
Surgical operation				
No	53(20.6)	6.20	0.005	1.00
Yes	204(79.4)	15.50		0.61(0.44-0.86)
Chemotherapy or radiotherapy				
No	31(12.1)	9.00	0.803	1.00
Yes	226(87.9)	13.30		0.94(0.59-1.51)

Abbreviation: MST, median survival time; HR, hazard ratio; CI, confidence interval.

### Genotype frequencies of genetic polymorphisms

Table 2 showed the genotype frequencies of four SNPs in 257 patients. All genotype frequencies of these four polymorphisms were found to be in Hardy-Weinberg equilibrium. The variant allele frequencies were 13.0% for *ERCC2 *751, 5.6% for *ERCC2 *312, 27.2% for *ERCC1 *118 and 34.6% for *XRCC1 *399 polymorphism. No associations were found between genotypes and age, tumor stage, surgical operation, chemotherapy or radiotherapy (data not shown).

**Table 2:** Genetic polymorphisms in DNA repair genes and survival of patients

Genotype	Patients(%)	MST(mon)	Log-rank P	HR(95%CI)	Adjusted HR(95%CI)^A^
*ERCC2 *Lys751Gln					
AA	194(75.5)	14.50		1.00	1.00
AC	59(23.0)	10.40	0.10	1.40(1.01-1.94)	1.32(0.95-1.84)
CC	4(1.5)	12.27		0.73(0.23-2.28)	0.79(0.25-2.48)
AC/CC^B^	63(24.5)	10.97	0.08	1.33(0.97-1.82)	1.27(0.92-1.75)
*ERCC2 *Asp312Asn					
GG	229(89.1)	13.23		1.00	1.00
GA	27(10.5)	12.40	0.05	0.95(0.60-1.49)	0.95(0.60-1.49)
AA	1(0.4)	4.40		8.25(1.12-60.60)	8.55(1.14-64.25)
GA/AA^C^	28(10.9)	9.83	0.96	0.99(0.63-1.54)	0.99(0.63-1.54)
*ERCC1 *Asn118Asn					
CC	140(54.5)	17.23		1.00	1.00
CT	94(36.6)	11.07	< 0.001	1.58(1.18-2.12)	1.48(1.10-1.99)
TT	23(8.9)	6.20		2.82(1.72-4.62)	2.67(1.62-4.40)
*XRCC1 *Arg399Gln					
GG	117(45.5)	19.10		1.00	1.00
GA	102(39.7)	10.40	< 0.001	1.41(1.05-1.91)	1.28(0.95-1.74)
AA	38(14.8)	9.23		2.33(1.56-3.47)	2.68(1.79-4.02)

Abbreviation: MST, median survival time; HR, hazard ratio; CI, confidence interval.

^A^HR adjusted for age, stage, surgical operation and chemotherapy or radiotherapy.

^B^Log-rank P and HR were calculated compared with wild genotype(AA) of *ERCC2 *751 polymorphism.

^C^Log-rank P and HR were calculated compared with wild genotype(GG) of *ERCC2 *312 polymorphism.

### Genetic polymorphisms and survival of patients

The associations between genotypes of four SNPs and survival of non-smoking female patients with lung adenocarcinoma were suggested in Table 2. Patients with CT or TT genotype at *ERCC1 *Asn118Asn showed significantly shorter survival time than those with CC genotype (11.07, 6.20 months versus 17.23 months) (Log-rank test, P < 0.001). In terms of *XRCC1 *399 polymorphism, the difference in the MSTs among patients with GG (19.10 months), GA (10.40 months) and AA (9.23 months) was statistically significant (Log-rank test, P < 0.001). However no associations were found between two SNPs of *ERCC2 *gene and the overall survival of patients.

**Figure 1:**
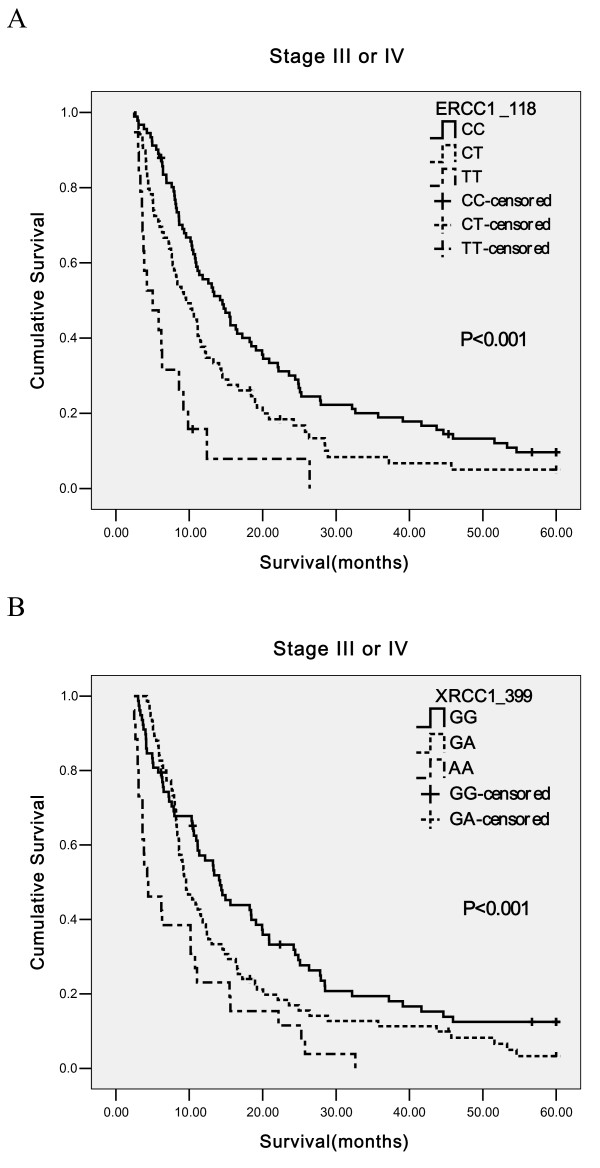
**Kaplan-Meier curves for patients with stage III-IV by (A) *ERCC1 *118 genotypes and (B) *XRCC1 *399 genotypes**.

In the further analysis, lung adenocarcinoma patients were stratified by stage. There were no significant differences in survival times of stage I-II patients with variant genotypes of four SNPs. For patients with stage III or IV, Kaplan-Meier analyses proved that individuals with heterozygous variant or homozygous variant genotype at *ERCC1 *Asn118Asn or *XRCC1 *Arg399Gln had shorter MSTs than those with wild genotype (Figure 1). The MSTs of patients with CC, CT and TT genotype at *ERCC1 *Asn118Asn were 14.53, 9.57 and 5.03 months and the difference was significant (Log-rank P < 0.001). For *XRCC1 *399 polymorphism, patients with GA or AA genotype lived shorter than those with GG genotype and corresponding MSTs were 9.57, 4.27 and 14.20 months(Log-rank P < 0.001). There were no differences in MSTs according to genotypes of *ERCC2 *751 or 312 polymorphism.

In the Cox regression model, after adjusting for age, tumor stage, surgical operation and chemotherapy or radiotherapy, variant genotypes of *ERCC1 *118 or *XRCC1 *399 polymorphism were associated with higher risks of death for non-smoking female patients with lung adenocarcinoma (Table 2). With the CC genotype at *ERCC1 *Asn118Asn being the reference, the HR for CT genotype was 1.48 (P = 0.009) compared to 2.67 (P < 0.001) in the TT genotype. In terms of *XRCC1 *399 polymorphism, we found that compared with those carrying GG genotype, the HRs were 1.28 (P = 0.109) and 2.68 (P < 0.001) for individuals with GA and AA genotype, respectively.

In addition to evaluating the genetic polymorphisms separately, we studied the association between patients' survival and the total number of variant alleles of *ERCC1 *and *XRCC1 *polymorphisms (Table 3). In the double homozygous group (0 variant allele), the overall MST was 25.10 months. As the number of variant alleles increased, the MSTs decreased to 13.07, 9.27, 6.30 and 3.87 months. The Log-rank test was statistically significant (P < 0.001). The HRs for individuals with 1, 2, 3, 4 variant allele(s) were 1.52, 2.33, 2.98 and 9.24 compared with those carrying 0 variant allele. Besides, the effect was also conspicuous for the patients with stage III-IV (Log-rank P < 0.001) but not significant for those with stage I-II (P = 0.145).

**Table 3:** Genetic polymorphisms in combination of *ERCC1 *and *XRCC1 *and survival of patients

Stage	Combined genotype	Patients(%)	MST(mon)	Log-rank P	HR(95%CI)^a^
all	0 variant	67(26.1)	25.10		1.00
	1 variant allele	100(38.9)	13.07		1.52(1.04-2.22)
	2 variant alleles	58(22.6)	9.27	< 0.001	2.33(1.54-3.50)
	3 variant alleles	26(10.1)	6.30		2.98(1.76-5.04)
	4 variant alleles	6(2.3)	3.87		9.24(3.50-24.44)
I and II	0 variant	26(33.3)	45.80		1.00
	1 variant allele	30(38.5)	22.27		1.98(0.86-4.58)
	2 variant alleles	14(17.9)	11.60	0.145	2.81(1.10-7.13)
	3 variant alleles	6(7.7)	--^b^		1.41(0.26-6.75)
	4 variant alleles	2 (2.6)	17.27		9.05(1.04-78.76)
III and IV	0 variant	41(22.9)	19.10		1.00
	1 variant allele	70(39.1)	11.17		1.54(0.98-2.39)
	2 variant alleles	44(24.6)	8.93	< 0.001	2.29(1.44-3.66)
	3 variant alleles	20(11.2)	5.83		3.69(2.06-6.62)
	4 variant alleles	4(2.2)	3.63		18.50(5.68-60.23)

Abbreviation: MST, median survival time; HR, hazard ratio; CI, confidence interval.

^a^HR adjusted for age, stage, surgical operation and chemotherapy or radiotherapy.

^b^MST for this genotype could not be calculated.

Furthermore we evaluated an interaction effect of the two SNPs on the survival of non-smoking female patients with lung adenocarcinoma. We found that individuals carrying both *ERCC1 *118 and *XRCC1 *399 variant alleles were at a higher death risk of lung adenocarcinoma than those with only one of them (adjusted HRs were 2.44, 1.79 and 1.64, respectively) (Table 4).

**Table 4:** Interaction of *ERCC1 *and *XRCC1 *polymorphisms on survival of lung adenocarcinoma

	*ERCC1 *CC		*ERCC1* CT/TT	
	
*XRCC1*	MST	HR(95%CI)^A^	MST	HR(95%CI)^A^
GG	25.10	1.00	13.30	1.79(1.16-2.76)
GA/AA	12.47	1.64(1.11-2.44)	8.60	2.44(1.63-3.65)

Abbreviation: MST, median survival time; HR, hazard ratio; CI, confidence interval.

^A^HR and 95%CI were calculated by Cox regression, with the *ERCC1 *wild genotype (CC) and *XRCC1 *wild genotype (GG) as the reference group;HRs were adjusted for age, stage, surgical operation and chemotherapy or radiotherapy.

Stepwise Cox proportional hazard analysis was used to study the relationship between factors including demographic characteristics, clinical features and genetic SNPs and survival of non-smoking female patients with lung adenocarcinoma. Four variables (stage, chemotherapy or radiotherapy, *ERCC1 *118 and *XRCC1 *399 polymorphisms) were included in the Cox regression model (Table 5).

**Table 5:** Stepwise Cox regression analysis on survival of lung adenocarcinoma

Variable	β	SEM	HR	95%CI	P value
stage (III+ IV vs. I+II)	0.661	0.102	1.94	1.59-2.36	< 0.001
chemotherapy or radiotherapy	-1.095	0.307	0.34	0.18-0.61	< 0.001
*ERCC1 *118 (CT/TT vs. CC)	0.473	0.142	1.60	1.22-2.12	0.001
*XRCC1 *399 (GA/AA vs. GG)	0.405	0.143	1.50	1.13-1.98	0.005

Abbreviation: SEM, Standard error of difference; HR, hazard ratio; CI, confidence interval.

β, regression coefficient.

## Discussion

In this study, we explored the relationship between SNPs of three DNA repair genes and survival of non-smoking female patients with lung adenocarcinoma in China. In WHO Western Pacific Region, lung cancer was the commonest cancer cause of death in women and the proportion of adenocarcinoma in lung cancer has increased quickly in both genders over the last few decades [14]. So we focused our study on lung adenocarcinoma in females who were requested to be non-smokers in order to control the confounding influence of smoking. This study showed that being advanced stage, without chemotherapy or radiotherapy, carrying variant genotypes at *ERCC1 *Asn118Asn or *XRCC1 *Arg399Gln were proved to be unfavorable prognostic factors for lung adenocarcinoma in Chinese non-smoking women. However, the associations have not been found between *ERCC2 *751 or 312 polymorphism and survival of lung adenocarcinoma.

The XRCC1 protein is considered to play an important role in both base excision repair and single-strand break repair. *XRCC1 *Arg399Gln polymorphism was the commonest one among more than 60 validated SNPs in *XRCC1 *gene and showed no major variations by ethnicity [15]. This polymorphism has been suggested to be a risk factor for the development of lung cancer [16-20]. In our previous study, a significant association between *XRCC1 *399Gln/Gln genotype and risk of lung cancer in Chinese non-smoking women was found [21]. In the present study, we found that non-smoking female lung adenocarcinoma patients with AA genotype at *XRCC1 *Arg399Gln had a shorter survival time (9.23 months vs. 19.10 months) and higher risk of death (adjusted HR = 2.68, 95%CI = 1.79-4.02) than those with GG genotype. It is consistent with other studies, although those results were obtained from the subjects of all genders, smoking status and histopathologic subtypes [22-24].

As for *ERCC1 *Asn118Asn polymorphism, we observed that the patients with CT or TT genotype showed a significantly shorter survival time than those with CC genotype (11.07 and 6.20 versus 17.23 months). In the multivariable Cox regression, *ERCC1 *118 variant genotype (CT/TT) remained prognostic factors of lung adenocarcinoma (HR = 1.60, P value = 0.001). To date, only a few studies have examined the relationship between *ERCC1 *polymorphism and survival of lung cancer, and they didn't control the influence of gender, smoking status and histopathologic subtypes [25-27]. In terms of chemotherapy response, a few studies suggested an association between *ERCC1 *Asn118Asn polymorphism and response to platinum-based treatment of lung cancer [25,27,28]. Although these studies have shown possible relationship between *ERCC1 *polymorphism and effectiveness of cancer treatment or survival of cancer patients, the biological effect of this synonymous SNP is unclear.

In our study, no associations were found between the overall survival and two SNPs of *ERCC2 *gene. This is consistent with other studies [22,25,29], but there are studies showing an opposite effect [24]. The explanation for these discordant results remains to be elucidated.

Many clinical features may play important roles in the survival of cancer patients. The multivariable Cox regression in this study showed that being advanced stage (III+IV) and without chemotherapy or radiotherapy treatment were independent unfavorable prognostic factor for lung adenocarcinoma in non-smoking female population. Surgical status wasn't included in the stepwise Cox model maybe because the surgical treatment is decided by the patients' conditions such as tumor stage and histopathologic subtypes, so the surgical status may be a dependent variable but not an independent prognostic factor.

Genetic polymorphisms as either prognostic or predictive biomarkers have many advantages, especially in the advanced cancer setting. First of all, the biological specimen for detecting SNP is easily to obtain. Second, the detecting method is simple, precise and practical. Finally, in the advanced cancer setting, diagnoses are made using specialized and mostly body-harmed method; otherwise SNP detecting can avoid these problems.

In conclusion, this study analyzed four SNPs in three DNA repair genes in relation to survival of non-smoking female patients with lung adenocarcinoma in China. The results suggested that besides clinical features such as tumor stage and chemotherapy or radiotherapy treatment, polymorphisms of *ERCC1 *Asn118Asn and *XRCC1 *Arg399Gln were associated with survival of patients. Because DNA repair is a complex system including many pathways and genes, larger studies with more genetic polymorphisms, even haplotypes, in different ethnic populations and the functional or biological relevance of these polymorphisms are needed to confirm our conclusions.

## Conclusions

Genetic polymorphisms in *ERCC1 *and *XRCC1 *genes might be prognostic factors in non-smoking female patients with lung adenocarcinoma.

## Competing interests

The authors declare that they have no competing interests.

## Authors' contributions

ZY, BZ, ML, and PG performed the analysis, interpreted the data and drafted the manuscript. BZ, QH, and ZC contributed to the design and coordination of the study and helped to draft the manuscript. XLL, RM, WB, SX, YJ, SX, YL, and XL participated in data collection, DNA isolation and interpreted the data. XX, XLL, ZY and MS participated in SNP genotyping and revised the manuscript. All authors read and approved the final manuscript.

## Pre-publication history

The pre-publication history for this paper can be accessed here:

http://www.biomedcentral.com/1471-2407/9/439/prepub
